# On the Role of Anisotropy of Membrane Components in Formation and Stabilization of Tubular Structures in Multicomponent Membranes

**DOI:** 10.1371/journal.pone.0073941

**Published:** 2013-09-16

**Authors:** Nataliya Bobrovska, Wojciech Góźdź, Veronika Kralj-Iglič, Aleš Iglič

**Affiliations:** 1 Institute of Physical Chemistry, Polish Academy of Sciences, Kasprzaka 44/52, 01–224 Warsaw, Poland; 2 Faculty of Health Studies, University of Ljubljana, Zdravstvena 5, SI-1000 Ljubljana, Slovenia; 3 Laboratory of Biophysics, Faculty of Electrical Engineering, University of Ljubljana, Tržaška 25, SI-1000 Ljubljana, Slovenia; Weizmann Institute of Science, Israel

## Abstract

Influence of isotropic and anisotropic properties of membrane constituents (nanodomains) on formation of tubular membrane structures in two-component vesicle is numerically investigated by minimization of the free energy functional based on the deviatoric-elasticity model of the membrane. It is shown that the lateral redistribution and segregation of membrane components may induce substantial change in membrane curvature resulting in the growth of highly curved tubular structures.

## Introduction

The shape of lipid bilayers, cellular or artificial, strongly depends on composition and lateral distribution of membrane components [1]. In cell membranes as well as in multicomponent artificial membranes the aggregation/segregation of membrane components may occur under different physiological or non-physiological conditions [2]–[5].

Except for the sake of simplicity there is no a priori reason to consider membrane constituents/nanodomains to be isotropic [6 7] instead of anisotropic, which actually represents a more general approach [2], [5], [8]–[12].

Not only proteins and/or protein-lipid complexes but also lipid molecules should be in general considered anisotropic [12]–[14]. Thermal rotational motion of lipids around their vertical axes may lead to wrong conclusion that the average (effective) intrinsic shape of lipid molecules is axisymmetric, i.e. isotropic. The membrane lipids have two tails and in general anisotropic headgroups. The rotational states in the curvature field of the membrane have different energy (except for the planar and spherical membranes). Averaging over rotational degrees of freedom gives effective anisotropic intrinsic shape of lipids [15]. When the membrane components are modeled as anisotropic, it is possible to explain formation of experimentally observed, transient, energetically stable, narrow necks (pores) connecting the fused vesicles to the target membrane. Such shapes may result from orientational ordering and lateral redistribution of membrane constituents/nanodomains [16].

Coupling between the cell/liposome shape and non-homogeneous lateral distribution [17] of membrane components may originate from the tendency of membrane components to find/induce the optimal configuration (optimal membrane curvature) with respect to the intrinsic shape of membrane components. It was indicated in different theoretical and experimental studies [5], [8]–[10], [12], [13], [18]–[22]. that the generation and stability of the lipid bilayer tubes in the cellular and artificial multicomponent membrane systems in the absence of *elongated inner stiff supporters*, e.g. microtubules [23]–[27] or *external pulling forces*, such as, optical tweezers [28], [29] or motor proteins (kinesin, dynamin) [29], [30], can be explained by the presence of membrane elements (nanodomains) and attached proteins with *anisotropic* properties. As for example, the membrane attached crescent shaped BAR domain proteins have *clearly anisotropic shape* and therefore their energy depend on their local orientation or statistically averaged local orientation, depending on the local curvature of the membrane [5], [20], [31]. The nanodomain can be a macromolecule which is partially or fully embedded into membrane bilayer (such as multi-anchor polymers [32]), membrane attached proteins plus interacting lipids, a small protein-lipid cluster or a small cluster of different kind of lipids etc. The membrane is then considered as the self-assembly of nanodomains. The area of a single nanodomain can be in general much larger than the area of a single lipid molecule. The intrinsic shape of a nanodomain and a single lipid can be modeled within the framework of the deviatoric elasticity model with the appropriate choice of two principal intrinsic curvatures 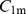 and 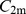 [15], [33].

**Figure 1:**
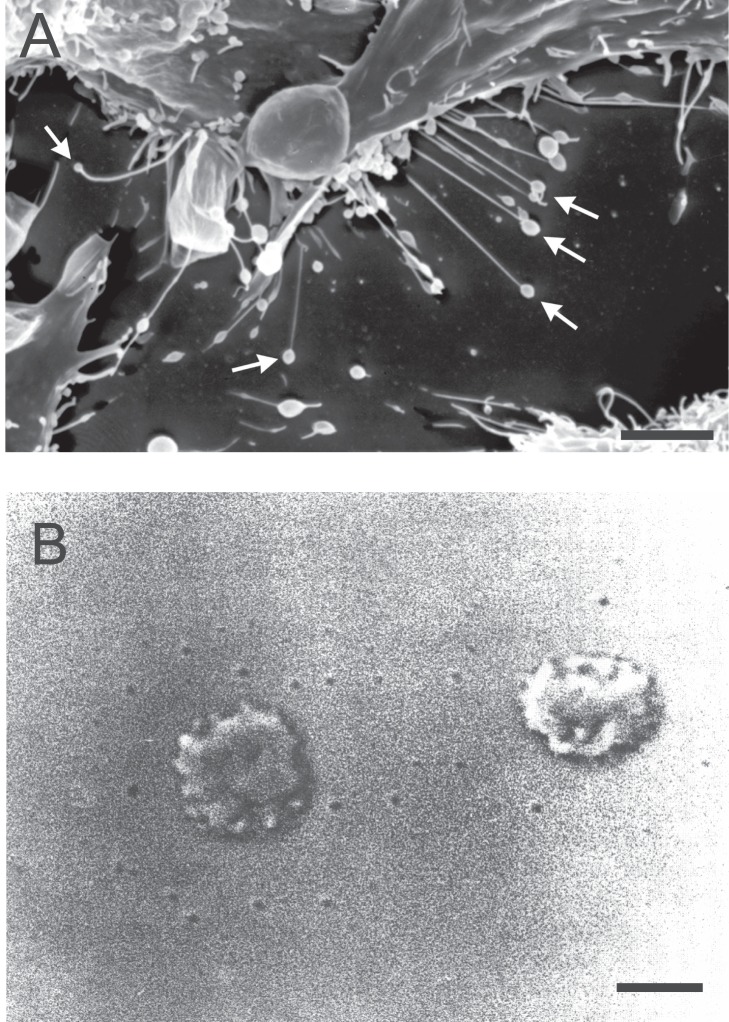
(A) Scanning electron micrograph of membrane nanotubes of RT4 urothelial cancer cells. Some of the nanotubes have spherical vesicles at their free tips (indicated by the arrows). Bar = 10 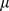
m (adapted with permission from [49].) (B) Vesiculation in human red blood cells. Note the exovesicles located around the parent red blood cells. Tethers are not visible in the figure. Bar = 3 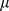
m (adapted with permission from [26]).

The aim of this work is to study the influence of anisotropy of membrane nanodomains on the shape transformations and lateral segregation of membrane components in two-component axially symmetric vesicles of fixed topology. The special attention is devoted to the stability and growth of tubular membrane structures with thin tubular protrusions having small spherical vesicles at their free tips (Fig. 1) induced by accumulation of anisotropic membrane components in tubular membrane regions.

## Results and Discussion

The model vesicles are built up by two components (A and B) ant their shapes are obtained numerically by the direct minimization of the free energy functional of the membrane under the constraints of constant vesicle surface area 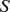 and volume 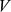 and a constant number of A type constituents/nanodomains, i.e. at constant total relative concentration of A component, 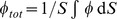 [24], [34]. The dimensionless reduced volume is defined as 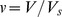 (the ratio of the volume of the vesicle to the volume of a sphere 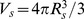 with the same surface area) where the radius 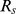 defines the unit length. The calculations were performed for vesicles with rotational symmetry, where the shape profile of the vesicle was described by the function 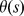 and the distribution of components on the vesicle surface by the function 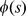 (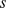 is the arclength of the profile). 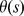 and 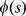 were calculated numerically by the minimization of the free energy functional [7], [24]. The minimization procedure and the detail description of 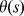 and 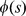 is given in the Methods section.

In this work, we have investigated under what conditions the formation of thin tubular structures is favorable. The special examples of such systems observed in experiments (Fig. 1), i.e. the cells with thin tubular protrusions having small spherical vesicles at their free tips, are the main subject of this work, not thoroughly studied in our previous work [22].

The calculations were performed for different values of the bending rigidity for each component, 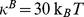, 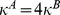. The value of 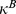 is characteristic for lipid domains, the value of 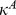 characterizes the domains of lipid membranes with attached macromolecules, where the thickness of the membrane is relatively large [35]–[37]. In the model, the bending rigidity depends on the local concentration of macromolecules, 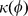 [38]. The function describing the local bending rigidity 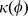 is defined in the Methods section. It has been assumed that the surface area of the nanodomain, 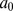, was of order of 100 nm^2^. The chosen vesicle radius in the calculations was of the order of 250 nm.

**Figure 2:**
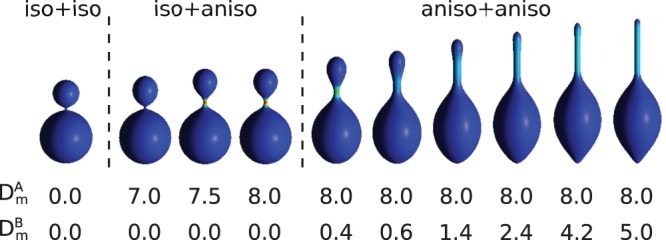
The shapes of the vesicle composed of (iso+iso), (iso+aniso), and (aniso+aniso) components for 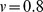, 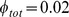, 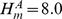, 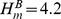, 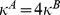.

When both components are isotropic, the vesicle is composed of small spherical beads connected by narrow passages, such as the first vesicle in Fig. 2. When one or two components are anisotropic we can obtain shapes in which thin tubular structures are formed. It is important to note that so far the formation of thin cylindrical protrusions which are attached to larger spherical vesicle has not been predicted in the models in which the anisotropy of the components is not taken into account [18], [22].

In the membrane systems encountered in the nature a small number of membrane components (minority) is usually strongly anisotropic, while a much larger number of membrane components (majority) is considerably less anisotropic, or isotropic. The anisotropic BAR domain proteins attached to the bilayer membrane [31], [39] are a typical example. For simplicity reasons we examine a single strongly anisotropic membrane component as a minority component, while the rest of the membrane is considered as composed of an isotropic component of a single type. We study the effect of concentration, reduced volume, and intrinsic mean curvature of an anisotropic membrane component on the formation of thin membrane tubular protrusions with a small vesicle at its tip. In the model, the local concentration of the components has an influence on the shape of the membrane, but also the curvature of the membrane determines the local distribution of the components [7], [40]. Thus, the vesicle shape and the local concentration of the components is determined by these two effects. If the distribution of the components did not depend on the shape of the vesicles, the components would be uniformly mixed in order to maximize the entropy. It has to be noted that we do not consider phase separated mixtures [41]. The segregation of components on the vesicle surface is due to the curvature gradients and the difference of the intrinsic curvatures of the constituents.

**Figure 3:**
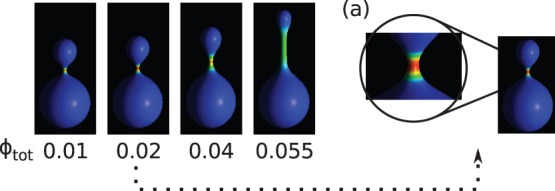
The vesicle shapes for different concentration, 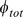, of anisotropic component, and for 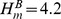, 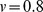, 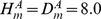, 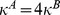. The inset (a) shows the configuration with almost total segregation of components for 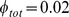. The anisotropic component is accumulated in the neck area.

The cylindrical protrusions are formed when at least one component is anisotropic. Such behavior is demonstrated in Fig. 3, where the total concentration is varied from 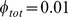 to 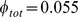.

It is interesting to note that very small amount of the anisotropic component is enough to induce the formation of the cylindrical protrusion. Moreover, the length of the protrusion depends on the concentration. This is due to the separation of the components in the membrane, where the anisotropic components are located mainly in the tubular part which has a very small surface area compared to the rest of the vesicle. It is interesting to note, that total component segregation was observed for 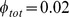. At this concentration almost all anisotropic component was accumulated in the neck of the vesicle. The possibility of accumulation of the components in a small area may be important for some macromolecules, since they are biologically active only at sufficiently high concentration.

**Figure 4:**
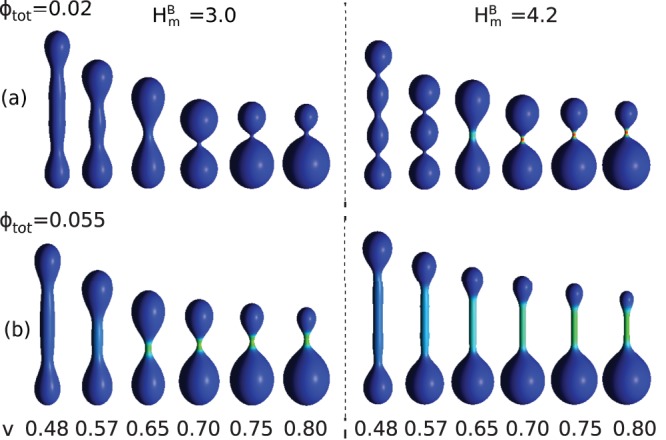
The vesicle shapes for different values of the reduced volume, 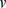, and for (a) 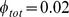, 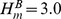 (left), 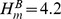 (right), 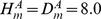, 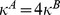 and (b) 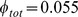, 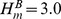 (left), 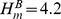 (right), 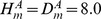, 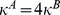.

The calculations presented in Fig. 3 were performed for constant reduced volume 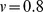. For such a reduced volume we observe the shapes without up-down symmetry, but for smaller values of the reduced volume the shapes with up-down symmetry are stable, as presented in Fig. 4. Moreover, the smaller the volume the more mixed are the components, and the cylindrical protrusions are no longer stable.

The complete mixing was observed for small concentration of the anisotropic component, 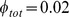, which for small reduced volume, 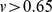, results in pearl-like shapes with up-down symmetry. The increase of the concentration of anisotropic component stabilizes longer and wider tubular structures.

**Figure 5:**
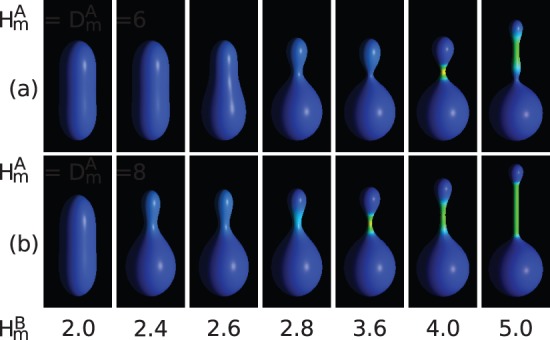
The vesicle shapes for different values of the mean curvature of isotropic component, 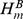, and for 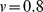, 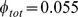, 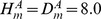, 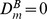, 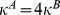: (a) 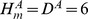 and (b) 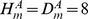.

The anisotropy of one of the components is not however a sufficient condition for the formation of the tubular structures. We have also observed that cylindrical protrusions may be induced by changing the properties of the isotropic component. It is demonstrated in Fig. 5 that when the intrinsic mean curvature of the isotropic component is increased (for fixed reduced volume) the cylindrical protrusions are formed and their length increases with the increase of the intrinsic mean curvature of the anisoropic component.

**Figure 6:**
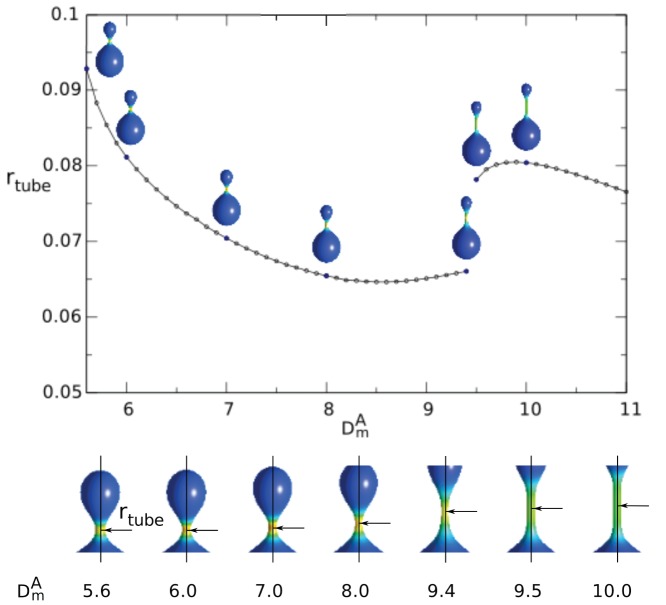
The change of the radius of the tubular region of a vesicle as a function of the intrinsic curvature of the anisotropic component, 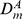, for 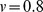, 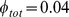, 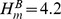, 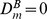, 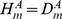, 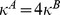. The arrows in the lower panel indicate the position on the surface of the vesicle where the radius of the tubule was measured.

In the systems in which the cylindrical tubules are created when the proteins (BAR domain proteins, epsin) are adsorbed at the membrane surface, the radius of the tubule is determined by the intrinsic curvature of the protein [42], [43]. In Fig. 6 we show that there is a strict relation between the intrinsic curvature, 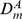, of the anisotropic component and the radius of the tubule. For smaller values of the intrinsic curvature, 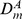, the cylindrical tubule is not well developed yet. At 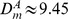 there is the transition to the well developed cylindrical tubule. Apart from the values of 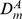 in the vicinity of the transition value 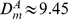 we can see that for the well developed cylindrical tubes (for 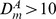) the radius of the tubular protrusion decreases linearly with increasing 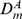. Thus, the results of our theoretical calculations are in qualitative agreement with the experimental predictions showing that the membrane tubular protrusions induced by the membrane bound anisotropic molecules (such as highly anisotropic BAR domain-containing proteins [5], [20], [31]) with larger intrinsic curvature radius (corresponding to smaller 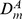 in our notation) generally have larger diameters than do those formed by the molecules characterized by smaller intrinsic curvature radius (i.e. larger 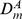) [5], [44].

### Conclusions

We have shown that accumulation of anisotropic components may lead to the formation of thin tubular protrusions. The anisotropy of components is a necessary condition for creation of the *stable* tubular protrusions. When the components are isotropic such cylindrical structures may be created only when some external force is applied. For example when membrane is pushed by growing microtubules or pulled by molecular motors. The width of the tubes depends on the intrinsic curvatures of anisotropic components. When the membrane is composed of isotropic components the *stable* protrusions which are created without any external force are built of a series of connected beads.

## Methods: Theoretical model and parametrization

In the model the membrane is composed of two components A and B which can be either isotropic or anisotropic and are characterized by the intrinsic principal curvatures 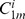, 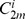 (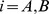). The free energy functional is composed of the (anisotropic) bending energy 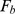 and the free energy associated with the entropy of mixing 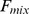:
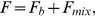
(1)where the membrane bending energy is given by [33], [45], [46]

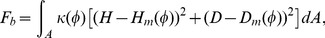
(2)while 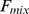 is [2], [46]

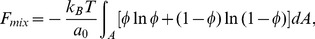
(3)where 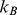 is the Boltzmann constant, 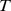 is the absolute temperature, 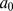 denotes the area of a single nanodomain, 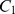 and 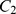 are the membrane principal curvatures, 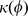 is the bending rigidity, 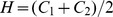 is the membrane mean curvature, 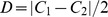 is the membrane curvature deviator, 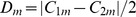 is the intrinsic nanodomain curvature deviator and 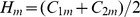 is the intrinsic nanodomain mean curvature, 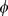 is the local relative concentration of the component A. The integral is taken over the whole surface of the vesicle membrane. The model parameters playing a crucial role in the vesicle shape transformations are: the total relative concentration (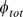) of the component A (the total relative concentration of the component B is 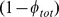), the bending rigidity of i-th component 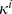, the nanodomain intrinsic mean curvature, 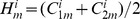, and the nanodomain intrinsic curvature deviator, 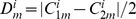. The components can be either anisotropic or isotropic. A component is considered as isotropic when its intrinsic deviatoric curvature is zero, 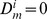. The properties of anisotropic components are defined by setting the intrinsic devatoric and the mean curvature equal 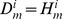.

For simplicity we assume linear dependence of the bending rigidity 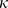 [47], the nanodomain intrinsic mean curvature 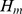 and the deviator 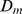 on the local relative concentration of the component A (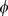):
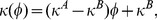
(4)

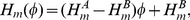
(5)

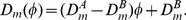
(6)


**Figure 7:**
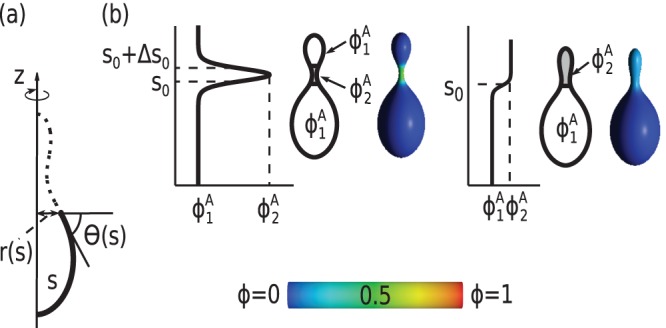
(a) Schematic representation of the parametrization of the vesicle shape. 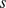 is the contour length, 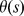 the angle between the tangent and the horizontal line, and 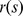 the distance from the 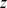-axis. (b) Color map shows the relative concentrations of anisotropic component, A, on the vesicle surface. Red color denotes high concentration of A component, blue color denotes high concentration of B component, green color denotes similar concentration of A and B component. 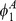 and 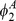 are the minimal and maximal local concentrations of the component A.

The contour of the vesicle is parametrized by the angle, 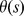, which is the function of the arclength of the contour (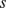) and is defined by the tangent line to the vesicle profile and a horizontal line, which is perpendicular to the axis of rotation (see Fig. 7a) [24], [34]. In this parametrization, the infinitesimal area element is given by 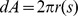, where 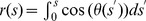 is the distance from the rotation axis. The principal curvatures are given by 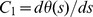 and 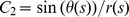 [34], [48].

The ansatz for the local relative concentration of the component A has the form :
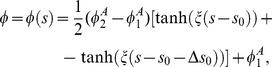
(7)where 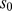 is the position of the boundary between the region rich in the component A and the region rich in the component B, 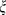 is the slope of the concentration profile at 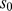, 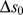 is the distance between the inflection points of two hyperbolic tangents. The validity of Eq.(7) assumes the division of the vesicle surface into two regions, which are characterized by the minimal and maximal local concentrations of the component A, 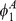 and 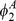, respectively (see also Fig. 7 b)

In numerical calculation we have to find both the function 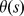 and the function 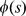 for which the functional (1) is minimized. In the minimization procedure the function 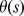 is expressed as a Fourier series. When the function 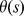, in the form of the Fourier series is plugged into the equation (1), the functional minimization can be replaced by the minimization of the function of many variables. The functional (1) becomes the function of many variables which are the amplitudes in the Fourier series and the length of the shape profile. Since the functional is minimized with respect to the shape and the concentration profile, a few additional variables 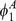, 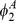, 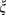, 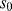, and 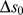 which determine the concentration profile, are also used in the minimization [7], [34], [40].
